# State-of-the-Art of the Nutritional Alternatives to the Use of Antibiotics in Humans and Monogastric Animals

**DOI:** 10.3390/ani10122199

**Published:** 2020-11-24

**Authors:** Vittorio Saettone, Ilaria Biasato, Elisabetta Radice, Achille Schiavone, Domenico Bergero, Giorgia Meineri

**Affiliations:** 1 Department of Veterinary Sciences, School of Agriculture and Veterinary Medicine, University of Turin, Grugliasco, Largo Braccini 2, 10095 Torino, Italy; vittorio.saettone@unito.it (V.S.); achille.schiavone@unito.it (A.S.); domenico.bergero@unito.it (D.B.); giorgia.meineri@unito.it (G.M.); 2 Department of Agricultural, Forestry and Food Sciences, School of Agriculture and Veterinary Medicine, University of Turin, Grugliasco, Largo Braccini 2, 10095 Torino, Italy; 3 Department of Surgical Sciences, Medical School, University of Turin, Corso Dogliotti 14, 10126 Torino, Italy; elisabetta.radice@unito.it

**Keywords:** antibiotics, intestinal microbiota, human, monogastric animals, pets, dysbiosis, probiotics, prebiotics, postbiotics

## Abstract

**Simple Summary:**

Antibiotic resistance represents a worldwide recognized issue affecting both human and veterinary medicine, with a particular focus being directed towards monogastric animals destined for human consumption. This scenario is the result of frequent utilization of the antibiotics either for therapeutic purposes (humans and animals) or as growth promoters (farmed animals). Therefore, the search for nutritional alternatives has progressively been the object of significant efforts by the scientific community. So far, probiotics, prebiotics and postbiotics are considered the most promising products, as they are capable of preventing or treating gastrointestinal diseases as well as restoring a eubiosis condition after antibiotic-induced dysbiosis development. This review provides an updated state-of-the-art of these nutritional alternatives in both humans and monogastric animals.

**Abstract:**

In recent years, the indiscriminate use of antibiotics has been perpetrated across human medicine, animals destined for zootechnical productions and companion animals. Apart from increasing the resistance rate of numerous microorganisms and generating multi-drug resistance (MDR), the nonrational administration of antibiotics causes sudden changes in the structure of the intestinal microbiota such as dysbiotic phenomena that can have a great clinical significance for both humans and animals. The aim of this review is to describe the state-of-the-art of alternative therapies to the use of antibiotics and their effectiveness in humans and monogastric animals (poultry, pigs, fish, rabbits, dogs and cats). In particular, those molecules (probiotics, prebiotics and postbiotics) which have a direct function on the gastrointestinal health are herein critically analysed in the prevention or treatment of gastrointestinal diseases or dysbiosis induced by the consumption of antibiotics.

## 1. Introduction

The second half of the 20th century discovered the novel use of antibiotics as growth promoters for food in the human diet, while the 21st century saw onset and rapid increase in advanced microbial antibiotics [1]. Over the past half century, antimicrobial resistance has become a growing medical concern due to persistent, selective pressure from the widespread use of antimicrobials in humans, animals and agriculture [2,3]. In 2001, the European Union health minister recommended a more rational administration of antimicrobial agents in human medicine, with a series of specific measures aimed at curbing antimicrobial resistance spread. Furthermore, alerting this crisis, the World Health Organization (WHO) in May 2015 adopted a global action to deal with antimicrobial resistance. This plan underlined the need for an effective one health approach that requires the coordination of several sectors including human and veterinary medicine, agriculture, finance, environment and consumers [4]. Despite this, the resistance rate to different bacterial species increases each year (especially against *Staphylococcus* spp., *Enterococcus* spp., *Enterobacteria* spp., *Pseudomonas* spp. and *Acinetobacter* spp.) [4]. The multi-drug resistance (MDR) definitions from the European Centre for Disease Prevention and Control (ECDC), published in 2011, were mainly chosen to harmonize epidemiological surveillance data in all health contexts and countries [5]. The ECDC criteria define MDR as acquired nonsusceptibility to at least one agent in three or more antimicrobial categories [6], thus underlying a representative limitation in the use of antibiotics, especially in severe clinical settings [7]. Therefore, in recent years, the European community has invested hundreds of millions of euros in funding research in antimicrobial resistance study, thus confirming its medical and social relevance. Based on these considerations, the search for new antimicrobial remedies is of vital importance. The aim of this work is to describe the state-of-the-art regarding possible alternative therapeutic strategies to the use of antibiotics in humans and monogastric animals and their impact on intestinal microbial ecology.

## 2. Intestinal Microbiota: Definition, Characteristics and Factors Affecting Its Balance

### 2.1. Humans

The human microbiota is a diverse microbial ecosystem now identified as an integral part of the gastrointestinal tract (GI) [8,9,10]. Indeed, in the last 30 years, numerous studies have been focused on its qualitative and quantitative characterization [11,12,13,14]. The first investigation by Suau et al. and Wilson et al. showed high diversity of faecal microbiota in healthy patients, as in-depth phylogenetic analyses revealed that the vast majority of the observed rDNA diversity was attributable to hitherto unknown dominant microorganisms within the human gut [15,16]. Within the last few years, metagenomics has also offered new insights into microbial diversity of the dominant microorganisms [17,18], thus providing a clear definition of the human microbiome. In particular, focusing on quantification, human intestinal microbiota is ten times higher than the prokaryotic cells of the organism, and their genetic heritage is clearly superior to human genome [19].

Tap et al. first demonstrated the existence of a bacterial “phylogenetic core” in the faecal microbiota of healthy adults [20]. The 16S rRNA gene sequences herein obtained confirmed that the predominant human faecal microbiota belonged to five phyla: Firmicutes (79.4%), Bacteroidetes (16.9%), Actinobacteria (2.5%), Proteobacteria (1%) and Verrucomicrobia (0.1%). Nevertheless, the composition of the human microbiota can be modified by some individual factors such as age, sex, nutrition status, lifestyle and circadian rhythms. With the aim of investigating all these variables in the human microbiota, the NIH Common Fund Human Microbiome Project (HMP) was established in 2008. So far, the HMP has developed metagenomic protocols for creating, processing and interpreting distinct types of high-throughput metagenomic data available to the scientific community [19]. Thanks to next-generation sequencing (NGS) of the small subunit ribosomal RNA (16s rRNA), it has been confirmed that Bacteroidetes and Firmicutes are the most abundant taxa in the intestinal microbiota of healthy adults [21,22], with approximately 500–1000 bacterial species belonging to *Bacteroides, Bifidobacterium, Eubacterium, Clostridium, Peptococcus, Peptostreptococcus, Lactobacillus* and *Ruminococcus* [23]. Specific changes can, however, be identified depending on the different stages of life. In particular, the way a baby is delivered influences postnatal microbial exposure. Indeed, babies born by vaginal delivery are exposed to vaginal microbes (*Lactobacillus* and *Prevotella* spp.) while the microbiome of babies born via C-section is dominated by *Staphylococcus*, *Corynebacterium* and *Propionibacterium* spp., which resemble microbial communities of skin [24,25]. During the first years of life, due to the introduction of solid foods, the composition of the intestinal microbiome becomes more complex, with a reduction of Bifidobacteria [26], when in puberty, major changes are driven by hormones, which determine the expression of genes related to development and growth [27,28]. In adulthood, the composition of the gut microbiome reaches a certain stability by increasing microbial count and complexity, with the increase in microbes belonging to the Lachnospiraceae and Ruminococcaceae families [29]. Ageing is accompanied by significant changes in lifestyle, such as decreased locomotion, nutritional changes and chronic consumption of medication, thus being accompanied by a general expansion of Bacteroidetes and reduction of Firmicutes phyla. Microbiome-associated metabolites (vitamins B7 and B12, and creatine) and their biosynthetic pathways are also reduced in ageing, thus contributing to muscle atrophy and frailty [22,30]. Noteworthily, gut microbiota in semi-supercentenarians (105–109 years old) is characterized by increased abundance of health-associated taxa like *Bifidobacterium, Christensenellaceae* and *Akkermansia* [31]. 

### 2.2. Monogastrics Animals

The definition of “intestinal microbiota” can equally be applied to both humans and the animals, since it is defined as “the usually complex mixture of bacterial populations that colonize a given area of the gastrointestinal tract in individual human or animal hosts that have not been affected by medical or experimental intervention or disease” [32]. However, considering the profound impact of the microbiome on human health, the majority of the available studies regarding the gastrointestinal microbiota and/or microbiome functionality has mainly been focused on humans [33]. Nevertheless, characterization of the intestinal microbiota has also been a topic of great research interest in monogastric animals since the 1970s. In particular, previous studies reported that the gastrointestinal microbiota of monogastric animals is composed primarily of bacteria, especially anaerobic, Gram-positive bacteria. The main bacterial species have been estimated to range from 400 to 500, with the bacterial densities generally increasing from the proximal to the distal gut until the identification of 1010–1012 bacterial cells per each gram of colon content or faeces [34]. The recent adoption of NGS technologies instead of classical cultivation-based methods has further improved scientific knowledge about the bacterial diversity of the gastrointestinal tract, especially in monogastric livestock and fish species. The great attention given to livestock farming is a direct consequence of its primary goal, which is represented by optimization of the feed efficiency and growth performance of the animals. This scenario has created a clear separation between humans and animals in terms of microbiota research, preparing the way for the rise of the concept of “gut health”.

Kogut and Arsenault firstly introduced the “gut health” concept as “the new paradigm in food animal production”, underlining its vital importance to the performance of production animals and defining it as synonymous with animal health within animal production industries [35]. Although a clear scientific definition of “gut health” is still lacking, six major components related to gastrointestinal functionality have recently been proposed [36]. Among them—including diet, effective digestion and absorption, functional immune status, gut mucosa, and neuroendocrine and motor function of the gut—a normal and stable microbiota has a key role because a symbiotic equilibrium between the intestinal tract and the microbiota is fundamental for maintaining the welfare and performance of the animals [36]. In particular, the gut microbiota benefits the host by providing useful nutrients (i.e., short chain fatty acids (SCFAs)) from poorly digestible dietary substrates (i.e., non-starch polysaccharides) and by modulating the development and functionality of the digestive (in terms of mucosal morphology and mucin dynamics) and the immune (in terms of antimicrobial peptides and pro-inflammatory cytokines production) systems [37].

As a consequence of the development of the “gut health” concept, the gut microbiota of the main monogastric livestock (such as poultry, pig, rabbit and fish) species has been extensively characterized, also sharing similarities among each other. Indeed, Firmicutes and Bacteroidetes bacterial phyla represent the intestinal core microbiota in all monogastric livestock (poultry [38], pig [39] and rabbits [40]) and fish species [41]. Proteobacteria is another dominant phylum in poultry and pig [38,39], but it is mostly identified in fish species, along with Actinobacteria and Fusobacteria. Within the Firmicutes phylum, Lactobacillaceae, Clostridiaceae and Ruminococcaceae represent the dominant bacterial families in the monogastric livestock species, with *Clostridium, Ruminococcus* and *Lactobacillus* being also the most abundant species in poultry [38], pig [39] and rabbit [40]. On the contrary, Carnobacteriaceae and Leuconostocaceae are characteristic of fish gut microbiota [42,43] Similarly, the Bacteroidetes phylum comprises Bacteroidaceae and *Bacteroides* as predominant members of the intestinal microbiota of the monogastric livestock species [38,40], while the fish gut microbiota mainly displays the Flavobacteriaceae family [44]. As a final aspect to consider, within the phyla Proteobacteria, Enterobacteriaceae, Campylobacteriaceae and Helicobacteriaceae represent the dominant bacterial families in the monogastric livestock species, with *Helicobacter* and *Actinobacillus* being also the most abundant species in poultry [38] and pig [39]. On the contrary, Aeromonadaceae and Vibrionaceae are characteristic of freshwater and marine fish intestinal microbiota, respectively [45,46].

Differently from the monogastric livestock and fish species, research about the gut microbiota in domestic animals (such as dogs and cats) share more similarities with the human field, since pets are nowadays considered as genuine family members and, as a consequence, all the factors potentially promoting their longevity (including the microbiome) are topics of great interest. Both canine and feline gut microbiota are dominated by the already mentioned Firmicutes, Proteobacteria and Bacteroidetes phyla [47]. However, the poor statistical power due to small sample sizes of pet studies has led to some inconsistencies in taxonomy findings across the studies [47]. Nevertheless, Alessandri et al. recently identified *Fusobacterium*, *Prevotella* and *Bacteroides* as major bacterial genera in the intestinal microbiota of both dogs and cats [48].

## 3. Intestinal Dysbiosis in Human and Animals: The Role of Antibiotics

### 3.1. Humans

The term “dysbiosis”, originally established by Metchnikoff to describe altered pathogenic bacteria in the gut [49], has also been defined as “qualitative and quantitative changes in the intestinal flora, their metabolic activity and their local distribution” [50]. In addition, antibiotics administration represents a cause of major alterations in normal gastrointestinal tract (GIT) [51] by inducing long-lasting changes in intestinal microbiota correlated with disease development [52]. The potential for an antimicrobial agent to influence the gut microflora is related to its spectrum of activity [51], pharmacokinetics, dosage [53] and length of administration [53]. Regarding the spectrum of activity, an antimicrobial agent active against both the Gram-positive and -negative organisms will have greater impact on the intestinal flora [51]. In terms of pharmacokinetics, the rate of intestinal absorption plays a fundamental role. In general, oral antimicrobials that are well absorbed in the small intestine will have a minor impact on the colonic flora, whereas agents that are poorly absorbed may cause significant changes. Parenteral administration of antimicrobial agents is also not free from these consequences, as some of these agents can be secreted in their active forms in bile or saliva, or from the intestinal mucosa, thus resulting in remarkable alterations in the colonic flora [54]. Microbiota alterations induced by a particular antibiotic might also be more severe in individuals who have been subjected to multiple courses of antibiotics. So far, antibiotics have been reported to increase Proteobacteria and Tenericutes but to decrease Bacteroidetes and Firmicutes. In particular, oral vancomycin may reduce the faecal microbial diversity by decreasing Gram-positive bacteria (mainly Firmicutes) and by increasing Gram-negative bacteria (mainly Proteobacteria) [55,56]. Indeed, potential biomarkers of antibiotic administration have been reported to be *Klebsiella*, *Parasutterella*, *Morganella*, unclassified_f_Enterobacteriaceae, *Ureaplasma* and unclassified_f_Ruminococcaceae (the majority of which belong to the Proteobacteria phylum). Based on these findings, Proteobacteria may be considered a potential, useful diagnostic sign of intestinal microbiota dysbiosis [55].

### 3.2. Monogastric Animals

Monogastric livestock farming has frequently been characterized by the indiscriminate use of antibiotics in order to control the development of diseases and to exploit their effects as growth promoters [57]. However, similar to that previously described for human patients, antibiotic administration may cause dysbiosis, a perturbation of the number and composition of the microbiota that affects physiological microbial balance [58]. Microbiota dysfunctions are generally associated with alterations in the digestive tract and in the metabolic processes, increase in nutrient absorption and downregulation of the immune response [57], with subsequent increase in pathogenic colonization and disease susceptibility [59]. Furthermore, the antibiotic-induced changes in microbiota composition can persist for months or years after cessation of antibiotic treatment, thus representing a long-term issue [58]. Another important aspect to consider is that misuse and overuse of antibiotics in livestock farming is closely related to the growing number of antimicrobial-resistant agents, thus raising important concerns about animal and human health [60] and necessitating the search for efficacious alternatives to their use [57].

#### 3.2.1. Poultry

Till now, several research studies have investigated the relationship between antibiotic treatment and gut dysbiosis in monogastric livestock species. Simon et al. first reported that the administration of antibiotics in the first weeks of life of laying hens may cause dysbiosis of the intestinal microbiota, thus potentially leading to alterations in immune development and, in turn, the immune competence of the birds [61]. Furthermore, zinc bacitracin administration has recently been reported to increase the abundance and the number of potential pathogens while decreasing those with beneficial functions in yellow-feathered broiler chickens [62]. These microbiota imbalances were also accompanied by lower microbial carbohydrate fermentation, higher microbial catabolism of amino acids and upregulation of pro-inflammatory cytokines after the antibiotic treatment [62]. The abovementioned research study confirms and extends the observations of Kumar et al. which identified higher abundance of *Campylobacter* spp. and *Salmonella* spp. as well as pro-inflammatory cytokines gene expression in bacitracin-fed chicks when compared to the control birds [63]. Tiamulin administration has also been reported to trigger intestinal dysbiosis in a laying hen model of gastrointestinal infection by *Brachyspira pilosicoli*, thus leading to higher susceptibility to pathogenic colonization and possibly causing relapse [64].

#### 3.2.2. Pigs

A similar scenario can also be highlighted in pigs, where weaning—the most critical part of their life cycle—is frequently associated with gut dysbiosis development. Antibiotic treatment may also further decrease microbial diversity and increase opportunities for pathogenic microorganisms to colonize and trigger diseases [60]. In particular, early antibiotic exposure in suckling piglets has been reported to influence intestinal microbiota, and carbohydrate and protein metabolism [59]. Connelly et al. also observed significant changes in a porcine model of ceftriaxone-mediated gut dysbiosis, where the intestinal microbiome was characterized by a reduced abundance of commensal species, outgrowth of colitis-causing species and increased frequency of antibiotic resistance genes [58]. A more recent porcine model of carbapenem-mediated intestinal dysbiosis confirmed the abovementioned findings, as commensal bacteria were undetectable after antibiotic treatment and multi-drug-resistant species were shown, instead, to have increased [65].

#### 3.2.3. Fish

As far as fish are concerned, the misuse of antibiotics, overdose and poor absorption after medication, as well as their high water solubility and bioactivity raise remarkable environmental issues in terms of ecological sustainability and health problems [66]. In addition to that, high concentrations of antibiotics used for treatment of bacterial diseases (florfenicol, oxytetracycline and sulfamethoxazole) have also been reported to cause severe microbiota dysfunctions in various fish species, such as Atlantic salmon [67], hybrid and Nile tilapia [66,68,69] and Zebrafish [70]. In particular, reduction [67] or increase [70] in microbial diversity and growth-depressing effects on some beneficial bacteria [66,68] were observed. Gut dysbiosis was also accompanied by upregulation of the intestinal pro-inflammatory cytokines [66,69,70] as well as altered gut histomorphology and tight junction proteins [66]. A high fat diet has also recently been reported to worsen the adverse effects of antibiotic administration on intestinal health in juvenile Nile tilapia in terms of gut microbiota disfunctions and altered intestinal histomorphology and tight junction proteins [69].

#### 3.2.4. Pets

Canine and feline intestinal dysbiosis can be described as an intestinal microbiota alteration of the composition and count (i.e., quantity of bacterial species) that is accompanied by a reduction in SCFA production [71,72]. Historically, bacterial proliferation of the small intestine has been used to describe qualitative and quantitative gut microbiota changes based on the juices of the duodenum and fasting cultures. Indeed, the bacterial population found in the proximal small intestine of several dogs has been reported to be substantially higher than that normally found in humans, where a bacterial count higher than 10^5^ CFU/g or ml in aspirates of the small intestine indicates bacterial overgrowth (SIBO). However, subsequent investigations have shown that healthy dogs could have bacterial loads that far exceed these established limits [73]. Therefore, SIBO is now a controversial definition and the terms antibiotic responsive diarrhoea (ARE) or small intestinal dysbiosis are preferred. More recently, the state of dysbiosis has been correlated to changes in the gut microbiota associated with gastrointestinal diseases—mostly affecting the large intestine—such as inflammatory bowel disease (IBD), granulomatous colitis, irritable bowel syndrome (IBS) [74], chronic enteropathy (CE), acute diarrhoea [75] and parasitic diseases (e.g., *Giardia duodenalis*) [76]. Although commensal bacterial microbiota deficiencies are linked to metabolic changes, it is not yet well investigated whether dysbiosis can be a cause or a consequence related to of gastrointestinal diseases. An overlap between these two scenarios seems reasonable, since inflammatory processes cause dysbiosis and recent functional studies have shown that dysbiosis, when present, may aggravate inflammation in genetically sensitive individuals [77]. In canine IBD, a proportional increase in bacterial genera belonging to the Proteobacteria phylum as well as a decrease in Fusobacteria, Bacteroidetes and members of Firmicutes has been observed in both the duodenum and the faeces [74,78]. Generally, there is a similarity in dysbiosis patterns between chronic and acute diarrhoea, even if some notable differences may be identified. Indeed, substantial increases in the populations of Fusobacteria have been observed in faecal samples from dogs with acute haemorrhagic diarrhoea [79], while *C. perfringens* overgrowth seems to characterize intestinal dysbiosis in chronic diarrhoea [74]. Unlike for those of dogs, only a few studies have focused their attention on faecal microbiota changes in cats with consolidated IBD. Janeczko et al. reported an increased duodenal count of enterobacteria in cats with IBD by fluorescence in situ hybridization, with these counts showing positive correlation with changes in mucosal architecture and cell infiltrates density [80]. An increase in *Desulfovibrio* spp. has also been observed [81], while Abecia et al. identified no differences between healthy cats and cats with IBD [82]. In a more recent study using 16S rRNA sequencing in cats with acute and chronic enteropathy but without a clear diagnosis, cats with chronic diarrhoea showed reduced proportions of Bacteroidetes, *Fecalibacterium* spp. and *Turicibacter* spp. and increased abundance of enterobacteria in their faecal microbiota similar to dogs with IBD [83]. However, no studies have clearly assessed whether dysbiosis patterns differ among the various forms of CE, food responsive enteropathy (FRE), ARE and IBD.

Antibiotic therapy, in association with dietary modifications, is commonly administered in dogs and cats with chronic enteropathy [84], the main antibiotic molecules represented by metronidazole and tylosin [85]. Metronidazole acts on bacteria and protozoa, while tylosin is useful dispensed during chronic tylosin-responsive diarrhoea that generally affects adults [85]. Moreover, specific enteropathies (such as boxer granulomatous colitis) usually require other antibiotic molecules belonging to the fluorquinolones class (e.g., enrofloxacin) [86]. Nevertheless, it is commonly known that antibiotic therapy may cause significant changes in the intestinal microbiota composition in either dogs or cats [77,87]. Indeed, microbiota alterations (i.e., increased abundance of organisms similar to *Escherichia coli*) may be still observed 14 days after tylosin withdrawal [88]. A more recent study in healthy dogs confirmed that the administration of tylosin induced dysbiosis but also demonstrated that eubiosis was not restored within 56 days after stopping tylosin, thus suggesting that the restoration of native microbiota is possible but not guaranteed [89]. Faecal bacterial diversity has also been reported to be reduced during the administration of oral metronidazole to healthy dogs for 14 days [90]. Similarly, oral administration of amoxicillin to healthy dogs for 7 days showed differences in faecal bacterial composition before and after, with several *E. coli* faecal isolates with greater resistance to more antibiotics during treatment [91]. In contrast, Kilpinen et al. demonstrated that dogs with tylosin-sensitive diarrhoea have shown an increase in *Enterococcus* spp. and other potentially probiotic bacteria (including lactic acid bacteria) in their faecal microbiota [92]. Therefore, it is thought that tylosin may have a certain probiotic action by exerting a selective increase in tylosin-resistant enterococci [92]. Nevertheless, even if these results appear interesting, there are still doubts about whether antibacterial resistance can pass horizontally from commensal or presumed probiotic bacteria to pathogenic bacteria that share the same intestinal environment [93]. Furthermore, the metabolic pathways through which antibiotics can alter intestinal homeostasis are still being investigated. 

## 4. Nutritional Alternatives to the Antibiotics and Effects on Intestinal Health: The Role of Probiotics

### 4.1. Humans

In 2013, the Food and Agriculture Organization of the United Nations (FAO) and the World Health Organization (WHO) scientific committees defined probiotics as “living microorganisms which, if administered in adequate quantities, confer a benefit for the health of the host” [94]. Indeed, probiotics are constantly administered to balance the homeostasis of the gut microbiota in order to maintain human intestinal health, to prevent and treat acute and chronic gastrointestinal diseases [95] by inhibiting the infiltration and growth of pathogenic bacteria in the GIT, and to prevent infections in patients with antibiotic-induced gut dysbiosis [96]. It has been suggested that probiotics may be beneficial and safe for the prevention of antibiotic-associated diarrhoea, as demonstrated in several randomized controlled trials (RCT). In particular, probiotics have been reported to be efficacious in the prevention of *C. difficile*-associated diarrhoea in both adults and children [97,98]. A previous review also suggested that the protective effects of probiotics as adjunct therapy may be used in the prevention of antibiotic-associated diarrhoea in outpatients of all ages, with probiotic intervention being capable of reducing its onset by 51% without any apparent side effects [99]. Indeed, several researches revealed that dietary *Lactobacillus* spp. and/or *Streptococcus* spp. supplementation reduced the incidence of antibiotic-associated diarrhoea in seniors (12.4% vs. 23.7% [100]), adults (6.9% vs. 14.2% [101]) and children (7.3% vs. 26.3% [101]).

In the past decades, probiotics have also gained increasing attention as a potential therapy against IBD—encompassing Crohn’s disease (CD), ulcerative colitis (UC) and indeterminate colitis (IC)—which can be differentiated by localization of the inflammation in the GIT [102]. However, despite probiotics having been used to induce remission and maintenance therapy in UC [103], a recent study indicated that probiotic supplementation in patients with IBD is a promising adjuvant treatment in UC but not in CD [102]. Vanderhoof et al. first observed that the administration of *Bifidobacterium longum* along with a prebiotic mix was capable of downregulating the expression of TNF-α and IL-1α pro-inflammatory cytokines and of attenuating inflammation in the intestine of IBD patients [97]. Dietary *Lactobacillus acidophilus* and *Bifidobacterium animalis* supplementation has also been reported to positively modulate the gut microbiota (in terms of increased *Lactobacillus* and *Bifidobacterium*) in IBD patients [99]. Furthermore, Yang and Yu reported that CD patients administered with a mixture of *Lactobacillus*, *Bifidobacterium* and *Streptococcus thermophilus* probiotics were characterized by a reduction in the mucosal inflammatory cytokine levels and severe recurrence of symptoms [104]. Considering that people with UC or CD, especially young people [105], are highly predisposed to develop colorectal cancer (CRC) [100], recent studies have demonstrated the potential role of probiotics in its prevention through modulation of the intestinal microbiota (in terms of increased *Lactobacillus* [106]) or metabolic activities [107]. The administration of *L. acidophilus*, *Bifidobacterium longum* and *Enterococcus fecalis* probiotics has also been reported to reduce diarrhoea incidence in patients affected by CRC [108]. Furthermore, selected strains of Clostridia may act as live biotherapeutic products to treat certain forms of IBD, allergy and other immune-inflammatory diseases thanks to the production of SCFAs and other metabolites [109].

### 4.2. Monogastric Animals

Similar to humans, the high occurrence of intestinal dysbiosis and the increasing development of antibiotic-resistance issues have progressively led to the search of efficacious alternatives to the use of antibiotics in monogastric livestock species and fish. Among the different agents that have been suggested to substitute antibiotics, probiotics (such as *Lactobacillus* spp., *Bacillus* spp., *Clostridium* spp. and *Saccharomyces* spp.) are widely considered to be one of the most effective because they preserve or restore the physiological microbiota, inhibit adhesion of pathogens to the intestinal wall, prevent gut inflammation and maintain the intestinal barrier function [110]. The positive effects of probiotic administration in monogastric animals in terms of improved growth performance, selection of beneficial bacteria, reduction of potential pathogens and stimulated immune response have extensively been characterized by several research studies and reviews, but there is limited information about their potential therapeutic or preventive role during the occurrence of specific gastrointestinal diseases (especially in relation to gut health implications).

#### 4.2.1. Poultry

Standard *Lactobacillus*-based probiotic administration (*Lactobacillus casei* 1.2435, *Lactobacillus rhamnosus* 621 and *L. rhamnosus* A4) has recently been reported to increase the relative abundance of beneficial bacteria and to decrease the relative abundance of harmful bacteria in the gut from Cherry Valley ducks infected with *E. coli* 77 [111]. Similar findings were also obtained when supplementing *Lactobacillus plantarum* K34 together with *Bacillus subtilis* MORI 91 and *Clostridium butyricum* M7 in *E. coli* O78-challenged broiler chickens in terms of increased total lactobacilli and total lactobacilli-enterococci populations in the intestinal microbiota and reduced isolation of *E. coli* 078 from liver and spleen [112]. El-Sharkawy et al. also recently reported that administration of different probiotic strains (such as *L. casei* ATTC334, *Bifidobacterium breve* JCM1192 and *Bifidobacterium infantis* BL2416) reduced *Salmonella* Typhimurium recovery from the caecal tonsils by competitive exclusion mechanism or by increased IFN-γ and TNF-α production [113]. Dietary *E. faecium* supplementation may also decrease the adverse effects of eimerian infection in experimentally infected broilers [114].

*C. butyricum* is a butyric acid-producing Gram-positive anaerobe that has been used as a probiotic to decrease clinical signs during IBD and antibiotic-associated diarrhoea in human patients [115]. In the wake of such positive outcomes, dietary supplementation of *C. butyricum* has been reported to improve the intestinal immune response, morphology and the digestive enzyme activities in broiler chickens challenged with *E. coli* K88 [116]. Zhao et al. also observed a decrease in *Salmonella enteriditis* colonization and reduced production of pro-inflammatory cytokines in liver, spleen and caecum from *S. enteriditis*-infected broilers [117]. In a more recent broiler model of necrotic enteritis, dietary administration of *C. butyricum* was also capable of modulating the immune response, of reducing the permeability and of decreasing *C. perfringens* counts in the gut [118].

*Bacillus* spp. represent another interesting probiotic for poultry due to their ability to produce heat-resistant spores and to their tolerance to the low pH, bile and enzymes encountered in the upper gastrointestinal tract of chickens [119]. In particular, dietary *B. subtilis* DSM 32315 has been reported to increase microbial alpha-diversity [120] and the relative abundance of beneficial bacteria as well as to decrease the relative abundance of *C. perfringens* in the gut from broiler chickens with necrotic enteritis [120,121].

*Saccharomyces cerevisiae* is a well-known yeast also exerting a probiotic influence in broilers by promotion of the metabolic processes of digestion and nutrient utilization. In particular, Massacci et al. has recently reported improved gut morphology and microbiota in *Campylobacter jejuni* challenged chickens after dietary *S. cerevisiae boulardii* CNCM I-1079 supplementation [122].

#### 4.2.2. Pigs

A similar scenario can also be highlighted in pigs, where the most investigated probiotics are represented by *L. rhamnosus*, *L. acidophilus* and *B. subtilis*.

Li et al. firstly recommended a safe threshold for using *L. rhamnosus* ACTT 7469 in clinical practice, since high doses may decrease the prophylactic benefits against potential enteric pathogens. Indeed, despite both the low and the high doses of probiotic administration ameliorating diarrhoea, reducing coliform shedding in faeces, increasing intestinal *Lactobacillus* and *Bifidobacterium* counts and attenuating the increase in gut pro-inflammatory cytokines in F4 (K88)-positive *E. coli*-infected piglets, high doses may negate the preventative effects by disturbing the established microbial ecosystem and by interfering with mucosal immune responses against potential enteric pathogens [123]. Zhang et al. also observed an attenuation of the expansion of Prevotellaceae NK3B31; the promotion of a symbiotic synergism of *Fusobacterium*, *Lactobacillus animalis* and *Propionibacterium*; and a restriction in chronic inflammation in the gut of *Salmonella enterica* serovar 4-infected pigs after administration of *L. rhamnosus* GG [124]. These findings partially confirm what was previously reported by the same research group, where dietary *L. rhamnosus* GG supplementation ameliorated the intestinal inflammation caused by *Salmonella* Infantis in a pig model, also excluding *Salmonella* from colonization of the jejunal mucosa and increasing the relative abundance of *Bifidobacterium* [125]. Nordeste et al. also investigated the efficacy of bioactive molecules isolated following the growth of *L. acidophilus* (called proteobiotics) in piglets challenged with *Escherichia coli* K88, observing increased abundance of *Lactobacillus* and reduced colonization by *Escherichia coli* K88 after prebiotic administration [126].

Similar to the effects of *L. rhamnosus* ACTT 7469 in *Escherichia coli*-infected piglets, different doses of a *B. licheniformis*–*B. subtilis* mixture have been reported to lead to contrasting outcomes. Indeed, pretreatment with low and moderate doses of the probiotic mixture attenuated the *E. coli*-induced expansion of selected bacterial species and increased the number of goblet cells in the gut of the infected piglets, while high doses disrupted colonic microbial ecology (in terms of increased abundance of Proteobacteria) and impaired goblet cell function in the ileum [127]. However, Luise et al. recently observed that dietary *B. subtilis* supplementation promoted the gut health of *E. coli* F4ac-infected piglets by reducing the Enterobacteriaceae level, favouring the upregulation of genes related to immunity and improving the amino acid metabolism and utilization [128].

#### 4.2.3. Fish

The use of probiotics (especially *Enterococcus casseliflavus*, *Rummeliibacillus stabekisii* and *Bacillus* spp.) in fish farming has primarily been focused on establishing effective prevention strategies (rather than therapeutic) towards *Aeromonas hydrophila* and *Streptococcus iniae* infections.

Dietary *E. casseliflavus* supplementation has been reported to significantly improve the host resistance to *S. iniae* infection in challenged rainbow trout by increasing the intestinal lactic acid bacteria and total viable aerobic counts and by stimulating the immune response of the animals [129]. Similarly, Tan et al. observed enhanced cumulative survival in Nile tilapia fed *R. stabekisii* after challenge with *A. hydrophila* and *S. iniae* thanks to stimulation of the immune response of the animals and positive modulation of their gut microbiota [130].

Administration of *Bacillus*-based probiotics (*Bacillus velezensis* TPS3N, *B. subtilis* TPS4 and *B. amyloliquefaciens* TPS17) has recently been observed to reduce the mortality rates in *A. hydrophila*-challenged Nile tilapia by enhancing both skin mucus and intestinal immune response, by improving the antioxidant defence, by enhancing the gut morphological and by positively modulating the intestinal microbiota [131]. Ahmadifard et al. also reported the lowest cumulative mortality in ornamental fish *Poecilia latipinna* pretreated with *B. subtilis*-enriched *Artemia* after *A. hydrophila* (BCCM5/LMG3770) challenging as a consequence of increased abundance of *Bacillus* in the gut [132].

#### 4.2.4. Rabbits

Unlike other monogastric animals, data regarding the potential therapeutic and/or preventive role of the probiotics against specific gastrointestinal diseases are very limited.

Ogawa et al. firstly reported that dietary *L. casei* strain Shirota supplementation enhanced local gut immune responses to Shiga toxin-producing *E. coli* (STEC) and Shiga toxins (Stx) 1 and 2 in STEC-infected infant rabbits, thus leading to the elimination of STEC, decrease in the Stx concentrations in the intestine and attenuation of the histological damage to gut mucus [133]. Administration of *L. casei* SABA6 strain in experimental rabbits challenged with *Escherichia coli* AZ1 has also recently been observed to attenuate the eruption of the intestinal epithelial cells, to reduce the incidence of diarrhoea and to stimulate the immune response of the animals [134].

#### 4.2.5. Pets

Despite the abovementioned definition of “probiotics” by FAO and WHO being worldwide accepted, health benefits associated with the administration of probiotics in pets are not strictly related to curing a specific disease. Therefore, it appears more appropriate to define probiotics as “microorganisms with the intention of improving the health of the host” [135], especially considering that their clinical effects have recently been characterized [136]. The EU Regulation 1831/2003 classifies probiotics as zootechnical additives, which are marketed only after specific authorization. At present, only four bacterial probiotic strains have been analysed by the European Food Safety Authority (EFSA) to evaluate safety and efficacy as feed additives in companion animals: two *E. faecium* strains (*E. faecium* NCIMB 10415 E1705 and *E. faecium* NCIMB 10415 E1707), *L. acidophilus* DSM 13241 25 and *B. animalis* [135]. As far as *E. faecium* NCIMB 10415 E1707 is concerned, the EFSA concluded that sufficient information was provided to consider its use as safe in pets and humans that have connections with the administered subjects. Indeed, *E. faecium* NCIMB 10415 E1705 has been defined as an unlikely cause of danger in target species even if overdosed, being also incapable of promoting the growth and spread of haemolytic and non-haemolytic *E. Coli* strains in dogs [135]. Many commercially available probiotics for pets contain Enterococci (mainly *E. faecium* or *E. fecalis*), which are natural inhabitants of the canine gastrointestinal tract. Specific strains can exert numerous benefits on the host such as probiotics, although Enterococci can also be pathogenic and have the ability to develop rapidly, to spread resistance to antibiotics and to promote the growth of potentially harmful microbes in animals and humans [137]. In relation to *L. acidophilus* DSM 13241 25, the EFSA revealed no safety concerns as the strain in question is sensitive to most antibiotics (excepted ciprofloxacin). The last examined probiotic strain, in 2012, was *B. animalis*, albeit the effects of *B. animalis* on health parameters related to the GIT in dogs were considered to have a questionable biological relevance, thus not allowing the EFSA to entrust the efficacy of this product. Adding to the abovementioned strains, in Europe and United States, though limited safety and efficacy data are available, the following strains are also marketed: lactobacilli (*L. acidophilus*, *L. casei*, *L. plantarum*, *L. paracasei*, *L. lactis*, *L. rhamnosus* and *L. salivarius*), bifidobacteria (*B. infantis*, *B. lactis*, *B. longum* and *B. bifidum*), *B. subtilis* or *coagulans* and, in some cases, yeasts (*Saccharomyces cerevisiae*) or fungi (*Aspergillus oryzae*).

From a general point of view, probiotics compete with potential pathogens by interposing with their adhesion to the intestinal mucosa or by promoting mucin release [138]. These mechanisms are believed to be specific of each strain, with some strains having greater adherence capacities (i.e., *L. rhamnosus*) and other strains being capable of increasing the adherence of pathogens to the intestinal mucosa [138]. In addition, probiotic bacteria can produce various antimicrobial substances, such as fatty acids, lactic acid and acetic acid [135,139]. To remain viable, probiotics must outlive stomach acidic environment and bile acids in order to successfully colonize the intestine, where they can bring advantages [140] by improving intestinal microbiota composition in terms of increased beneficial bacteria at the expense of unwanted ones [141,142]. However, it is important to underline that the production methods, the food carrier and the growth media can widely influence the original properties of probiotics, in particular, the adhesiveness capacity to the intestinal mucosa of canine lactobacilli [143]. Although lactobacilli represent only a small part of the canine gastrointestinal microbiota, they are still widespread, and several isolated strains, including *Lactobacillus* spp., demonstrate antimicrobial activity in vitro [144,145], survive and dominate the microbiota of the small intestine during feeding, and are capable of modifying the intestinal micro-ecosystem [146]. In addition, *L. fermentum* VET9A, *L. plantarum* VET14A and *L. rhamnosus* VET16A (administered alone or together) have been reported to show good adhesion to the canine intestinal mucosa and the ability to prevent the adhesion of common enteropathogens (such as *Enterococcus canis*, *C. perfringens* and *S.* Typhimurium) to the canine intestinal mucosa in vitro [147]. Strompfova et al. also observed increased faecal lactobacilli and enterococci, increased total serum proteins and lipids and decreased blood glucose as well as increased production of SCFAs and reduced proliferation of clostridia and selected Gram-negative bacteria (such as coliforms, *Aeromonas* spp. and *Pseudomonas* spp.) in the follow-up in dogs supplemented with *L. fermentum* AD1 (a probiotic originally isolated from the faeces of healthy dogs) [148]. The administration of canine *E. faecium* EE3 has been reported to reduce *Staphylococcus* spp. and *Pseudomonas*-like bacteria and to increase the concentration of *Lactobacillus* spp. [149]. Pascher et al. also observed that the administration of *L. acidophilus* DSM1324 increased faecal consistency, faecal dry matter, defecation frequency, faecal lactobacilli and bifidobacteria and reduced *C. perfringes* and *Escherichia* spp in German Shorthair Pointers dogs [150]. Furthermore, some authors think that bacilli represent better probiotics than lactobacilli, since they can sporulate and, in turn, may be more resistant to environmental stress and low pH [151]. However, independently of their survival, all the probiotics must be tested for their benefits in clinical situations. Indeed, Felix et al. found that *B. subtilis* C-3102 improved faecal consistency in dogs due to the decrease in faecal ammonia content, but the clinical relevance of this discovery is highly questionable, thus making the use of bacilli as probiotics not recommended [151].

Probiotics also appear to be a promising tool for alleviating gastrointestinal diseases in pets. Herstad et al. observed that the administration of a probiotic cocktail including *Lactobacillus farciminis*, *Pediococcus acidilactici* and *L. acidophilus* MA 64/4E was capable of reducing recovery time in acute self-limiting gastroenteritis in dogs [152]. As far as the acute canine gastrointestinal infections are concerned, dietary probiotic supplementation containing four strains of *Lactobacillus* (*L. casei*, *L. plantarum*, *L. acidophilus* and *L. bulgaricus*), three strains of *Bifidobacterium* (*B. longum*, *B. short* and *B. infantis*) and one strain of *Streptococcus salivaris* (*Streptococcus sulivarius subsp thermophilus*) has also been reported to increase the survival rates (90% vs. 70%) and to improve the clinical score and white blood cell/lymphocyte count in puppies with confirmed parvoviral enteritis [153]. Probiotics are not always capable of colonizing the GIT as a consequence of the competition with the intestinal microbiota that is already present [154]. Indeed, Garcia-Mazcorro et al. demonstrated that the abundant growth of *Enterococcus* spp. [155] and *Streptococcus* spp. promoted by oral integration of a synbiotic product (probiotic and prebiotic) was only transitory. Another study also showed a small growth of different species after the administration of *Enterococcus faecium*-based probiotic [154].

As a final consideration, some of the most recent studies involving probiotic molecules and their impact on intestinal health are reported (Table 1).

## 5. Other Nutrients Showing a Synergic Effect with Probiotics on Intestinal Health: Prebiotics and Postbiotics

### 5.1. Prebiotics

#### 5.1.1. Humans

Another useful approach to positively modulate the gut microbiota is represented by the administration of growth enhancing substrates—the so-called “prebiotics”—that can selectively be utilized by health promoting bacteria in order to stimulate their growth and the production of associated desirable metabolites. More recently, the term prebiotic was updated in December 2016 when the International Scientific Association for Probiotics and Prebiotics (ISAPP) described it as “a substrate that is selectively utilized by host microorganisms conferring a health benefit” [160]. Currently, a major prerequisite to the success of a particular prebiotic is that the target bacteria must be identifiable at a specific threshold. Indeed, if levels are below a certain threshold (as a consequence of ageing or antibiotic administration), the prebiotic may not be effective in increasing these desirable bacteria to confer the abovementioned health benefits [161]. Nevertheless, other important features need to be considered. While prebiotics can enable the growth of targeted health-promoting bacteria, more detailed microbiome analyses have, however, highlighted that these substrates are not always specific [160,162,163]. Furthermore, the benefits of prebiotics can be secondary, such as the production by health promoting bacteria of metabolic products that can inhibit the growth of enteric pathogens and/or attenuate their virulence [164,165,166].

Among the best characterized prebiotics, galactooligosaccharides (GOS), inulin and its oligomer fructooligosaccharides (FOS) appear to be the most promising ones [164,167,168]. In particular, the scientific community has mainly focused its attention on their effects on *Bifidobacterium* and *Lactobacillus*, since strains from these genera are recognized to confer health benefits to the host [169]. The GOS and FOS have overall been reported to improve the gut microbiota by increasing Bifidobacteria and by decreasing *E. coli* [170,171]. Nicolucci et al. also observed decreased abundance of Firmicutes and Ruminococcus as well increased abundance of Bifidobacteria in faecal samples after FOS-enriched inulin supplementation [172]. However, the effects of prebiotic therapy also depend on individual’s starting microbial profile. Indeed, in a study comparing FOS, sorghum and arabinoxylan, equally high SCFA production was observed in volunteers whose microbiota were dominant in fibre-utilizing *Prevotella*, while *Bacteroides*-dominated enterotypes showed different SCFAs levels in response to each fibre [173].

Pectin and pectic oligosaccharides (POS) have recently been identified as emerging prebiotics as they can selectively be utilized by certain members of the colonic microbiota such as *E. eligens* and *Faecalibacterium prausnitzii* [174,175]. The most common sources of pectin are citrus fruits and apple pulp, but it is also abundant in agricultural by-products such as sugar beet pulp. The POS can be obtained through depolymerization of pectin, and both the pectin and the POS are capable of escaping host digestion and, in turn, reaching the distal colon when consumed [176]. An in vitro assay of *F. prausnitzii* growth on different substrates firstly ascertained that growth was enhanced with apple pectin in the majority of cases [174,177]. Furthermore, lemon peel waste POS determined an increased level of *Fecalibacterium* and *Roseburia* spp. as well as lactobacilli [178].

Isomaltooligosaccharides (IMO) are also considered potential prebiotics. The IMOs are naturally found in foods such as honey or fermented foods such as miso and soy. Commercially available IMOs are composed of a mixture of α-(1,6) and α-(1,4)-linked glucosyl oligosaccharides. One of the glucose oligomers identified in IMOs is isomaltose, which is a major constituent of honey (thus giving IMOs a distinctive sweet honey taste). Breast milk is the natural, first nutritional source for newborns and provides oligosaccharides that promote the growth of desirable *Bifidobacterium* and *Lactobacillus* spp. in infant gut [179]. Indeed, the human milk oligosaccharide (HMO) and other nutrients in breast milk can act as substrates for bacteria in the gut, thus stimulating the growth of beneficial bacteria herein located. In particular, dietary HMO supplementation has recently been reported to modify the gut microbiota of adult volunteers by increasing the relative abundance of Actinobacteria (in particular *Bifidobacterium*) and by decreasing the relative abundance of Firmicutes and Proteobacteria [180].

Soybean oligosaccharides also seem to have a positive influence on *Bifidobacterium* levels and lactate production. In particular, soybean benefits are attributed to resistant starch (RS), which is a form of starch that cannot be degraded in the small intestine and, in turn, is capable of reaching the large intestine where it is fermented by the colonic bacteria [181]. Potato and maize RS have also recently been reported to modulate the gut microbiota of healthy volunteers in terms of increased *Ruminococcus bromii* and *Bifidobacterium* spp., with potato RS also leading to a greater SCFA production when compared to maize and inulin [182].

As a final consideration, synbiotics, which are a combination of prebiotics and probiotics meant to strengthen the effects of probiotics administered alone [183], also need to be mentioned, since they are capable of improving the survival of dietary live microbial supplements in the GI tract and of selectively stimulating the growth and/or activating the metabolism of health-promoting bacteria [184]. In particular, inulin has successfully been tested as a synbiotic component in the treatment of the ulcerative colitis [185,186].

#### 5.1.2. Monogastric Animals

As in humans, the synergic utilization of prebiotics with probiotics allows manipulation of the gut microbiota of animals, thus controlling the spread of bacterial infections and, in turn, avoiding the overuse of antibiotics [187]. In particular, the scientific community has recently focused its attention on the use of prebiotics (such as inulin, yeast cell products, mannanoligosaccharide (MOS), FOS, GOS, xylooligosaccharide (XOS) and IMO) as potential therapeutic or preventive feed additives during the occurrence of specific gastrointestinal diseases (especially in relation to gut health implications).

##### Poultry

Dietary live yeast (*S. cerevisiae*) and MOS supplementation has firstly been reported to attenuate the intestinal inflammation and barrier dysfunction as well as to improve gut morphology and microbiota in *Escherichia coli*-challenged broiler chickens [188]. Similarly, Rahimi et al. observed enhanced intestinal morphology as well as reduced shedding of *Salmonella* in turkey poults challenged with *Salmonella* and *Campylobacter* after administering a mixture of *S. cerevisiae* wall, MOS and a direct-fed microbial [189]. Dietary whole yeast cell product alone has also been reported to decrease faecal coccidial oocyst count and intestinal coccidial oocyst count, to increase the relative abundance of *Lactobacillus* and to attenuate the inflammatory response in *Eimeria*-challenged pullet and layer chickens [190].

As far as other prebiotics are concerned, Pourabedin et al. similarly showed reduced *S. enteriditis* counts, increased abundance of beneficial bacteria and a significant attenuation of the increase in pro-inflammatory cytokines in MOS- and XOS-fed young broiler chickens after infection with *S. enteriditis* [191]. The concomitant utilization of XOS, *Lactobacillus*-based probiotic and fermented soybean meal has also recently been reported to decrease gut *S.* Typhimurium colonization, to increase intestinal lactic acid bacteria, to improve gut mucosa morphology and to reduce the heterophil to lymphocyte ratio in *S.* Typhimurium-challenged young broiler chickens [192]. A significant reduction in *S. enteriditis* [187] and Typhimurium [193,194] loads as well as positive microbiome changes [194] were also observed in *Salmonella*-challenged white Leghorn chickens simultaneously administered with synbiotic products (FOS and probiotics) [187] or GOS [193,194]. Tarabees et al. finally reported increased total lactobacilli and total lactobacilli-enterococci populations in the intestinal microbiota along with reduced isolation of *E. coli* 078 from liver and spleen in broiler chickens fed diets supplemented with IMO and a commercial probiotic mix during *E. coli* O78 challenging [111].

##### Pigs

Apart from the abovementioned prebiotics, other unconventional, prebiotic-like feed additives have also been tested in pigs. The utilization of fructan-rich chicory roots has first of all been reported to reduce the intestinal *Ascaris suum* worms; to increase the intestinal *Trichuris suis* worms; to decrease the intestinal levels of *Enterobacter* and *Campylobacter*; and to increase the intestinal levels of Lachnospiraceae and Bifidobacteria in piglets experimentally infected with *Ascaris suum*, *Trichuris suis* and *E. coli* O138:F8 [195]. Myhill et al. also observed a synergic activity of inulin and *Trichuris suis* in a porcine model of *Trichuris suis* infection in terms of stimulation of the immune response, reduced abundance of Proteobacteria and Firmicutes, increased abundance of Actinobacteria and Bacteroidetes, and identification of the highest Bacteroidetes–Firmicutes ratio and the lowest gut pH [196]. The same authors recently confirmed that the administration of *Trichuris suis* together with the consumption of inulin may positively affect the gut health of the infected pigs by increasing the relative abundance of Prevotella and by decreasing the relative abundance of Proteobacteria [197]. Interestingly, dietary human milk oligosaccharide (HMO) and GOS-FOS mixture supplementation has also been reported to shorten the duration of diarrhoea, to enhance the immune system and to increase the relative abundance of butyrate-producing Lachnospiraceae in a porcine model of rotavirus OSU infection [198].

##### Fish and Rabbits

Unlike other monogastric animals, data regarding the potential therapeutic and/or preventive role of the prebiotics against specific gastrointestinal diseases in fish and rabbits are quite scarce.

The administration of a plant-based diet has firstly been reported to exert a prebiotic effect in *Yersinia ruckeri*-challenged rainbow trout by positively modulating their gut microbiota and immune response [199]. Similarly, Wang et al. observed enhanced levels of immune enzyme activities, increased proportions of *Bacillus* and *Lactococcus* and reduced levels of *Vibrio* in the intestine from sea cucumber fed symbiotic-based diets (containing the probiotic *Bacillus lincheniformis* WS-2 and the prebiotic alginate oligosaccharide) as prevention against *Vibrio* infections [200].

As far as rabbits are concerned, the administration of a prebiotic mixture (composed of S. cerevisiae cell wall, MOS and dried *S. cerevisiae* fermentation solubles) has recently been reported to reduce the faecal oocyst count and the intestinal endogenous stage counts in rabbits experimentally infected with *Eimeria* spp. [201].

##### Pets

Prebiotics are used in both healthy and sick dogs, being particularly effective in the management of obesity and gastrointestinal diseases. Indeed, Zentek et al. firstly observed increased Bifidobacteria and decreased *C. Perfringens* in faecal samples from healthy dogs fed diets containing 3% of chicory (1.5% of inulin) [202]. Similarly, the administration of FOS has been reported to increase Bifidobacteria and mineral digestibility in healthy dogs [203]. Alexander et al. also recently observed accelerated excretion of bile acids, an increase of Firmicutes and a decrease of Proteobacteria in obese dogs after inulin supplementation at 0.5–1% [204]. Furthermore, the administration of chondroitin sulphate and prebiotic-mix (consisting of RS, beta-glucans and MOS) has been reported to improve the serum oxidative stress markers (such as PON 1 and TAC) and cholesterol levels in IBD dogs [205]. Synbiotics may also represent useful products, as reduced diarrhoea rates may be identified in dogs after dietary supplementation with *E. faecium*, arabic gum and FOS [206].

Unlike dogs, research studies on the utilization of prebiotic substances in cats are very limited, as they consume few vegetables (and, in turn, carbohydrates) in their natural diet [207]. However, dietary FOS supplementation has previously been reported to increase the relative abundance of Bifidobacteria (alone) and lactobacilli (together with pectin) as well as to decrease the relative abundance of *E. coli* (alone) and *C. perfringens* (together with pectin) in cats [208].

As a final consideration, some of the most recent studies involving prebiotic molecules and their impact on intestinal health are reported (Table 2).

### 5.2. Postbiotics

#### 5.2.1. Humans

Postbiotics are bioactive compounds generally considered as secondary products of food quality microorganisms during a fermentation process, including microbial cells, cell constituents and metabolites. In detail, these functional fermentation compounds can be administered in combination with nutritional components to promote health or to increase the effectiveness of active microorganisms by transforming them into functional ingredients. Postbiotic effects derive from those microbial metabolites such as proteins, lipids, carbohydrates, vitamins, organic acids, cell wall components or other complex molecules produced in the fermented matrix [211,212]. The molecular mechanisms underlying the effects of postbiotics seem to be represented by the interaction between host and microbial products, thus activating the host’s immune system and, in turn, anti-inflammatory responses [213].

##### Short Chain Fatty Acids

The SCFAs (such as acetate, pronionate and butyrate) are important components of postbiotic products [214]. Primary SCFAs are well known to stimulate proliferative effects on colonocytes and to promote the absorption of water and sodium in the intestine. The most abundant SCFA detectable in human peripheral circulation is acetate, as propionate is metabolized by the liver during gluconeogenesis and butyrate is absorbed and used as a primary source of energy by colonocytes [215,216]. Furthermore, butyrate is linked to numerous beneficial health effects such as strengthening intestinal barrier function and mucosal immunity [217,218,219]. Furthermore, butyrate and, to a lesser extent, propionate are known to act as inhibitors of histone deacetylase, allowing them to exert anti-inflammatory and immune activity through the suppression of lamina propria macrophages and to cause the differentiation of dendritic cells from stem cells. bone marrow [216,220,221,222]. The SCFAs can also modulate cellular activity in the extracellular matrix via identifiable SCFA-specific G protein-coupled receptors on intestinal epithelial cells [223,224]. Another interesting aspect to underline is that the pathways for SCFA biosynthesis from the fermentation of indigestible fibres stimulate a bacterial cross-feeding complex involving several SCFA synthesis pathways to produce acetate, propionate and butyrate [216,225].

##### Antimicrobial Peptides (AMPs)

Postbiotic compounds might also play a crucial role in the inhibition of pathogens, since they contain the antimicrobial peptides (AMPs) [226]. The AMPs represent a wide range of molecules produced by the living organisms as a natural barrier against infections. In particular, the AMPs exhibit a wide range of activities against Gram-negative and Gram-positive bacteria, fungi, viruses and parasites. Among the AMP sources, bioactive agents derived from food proteins (mainly milk proteins) are particularly interesting because they are recognized as safe for both the humans and the animals [227,228].

Bovine and human milk contain a wide array of bioactive components such as the iron-binding glycoprotein known as lactoferrin (hLf in humans and bLf in bovine species). In both humans [229] and cows [230], the Lf concentration in milk changes according to the stage of postpartum lactation, with the highest concentrations being identified in the colostrum. This protein displays antibacterial, antifungal, antiparasitic, antiprotozoal [231,232], antiviral [233,234,235,236,237], immunomodulatory [238], anti-inflammatory, anti-catabolic and antioxidant activities [239,240,241,242], which overall contribute to the maintenance of homeostasis and the control of intestinal life-threatening diseases (especially in neonates [243]). In particular, the human Lf has been reported to show a very effective response against a wide range of bacteria, including *Streptococcus*, *Salmonella*, *Shigella*, *Staphylococcus* and *Enterobacter* spp. [244].

#### 5.2.2. Monogastric Animals

Similar to humans, postbiotics mainly consist of antimicrobial metabolites such as organic acids and bacteriocins that are capable of reducing the gut pH and, in turn, inhibiting the proliferation of opportunistic pathogens in the feed and gut of animals [245]. The postbiotics that are most commonly adopted as antibiotic alternatives in monogastric animals are represented by metabolites from *Lactobacillus* and, in the minority of cases, *Pediococcus*, *Enterococcus*, *Leuconostoc*, *Rhodotorula* and *Cetobacterium* (with the latter three being exclusively used in fish).

##### Poultry

The administration of postbiotics obtained from *L. plantarum* has firstly been reported to exert beneficial effects on the gut environment and immune system of healthy laying hens [246] and broilers [247,248] in terms of reduced pH [246], increased abundance of lactic acid bacteria [246,247] and *Bifidobacteria*, decreased abundance of Enterobacteriaceae [246,247] and *E. coli* [248], increased production of propionic [245] and acetic acids [246,247], and downregulation of pro-inflammatory cytokines [248]. Humam et al. also observed improved intestinal morphology and microbiota as well as the immune system in broilers under heat stress [245]. The administration of postbiotics obtained from *P. acidilactici*, *L. reuteri*, *E. faecium* and *L. acidophilus* has also recently been reported to improve the lesion scores, *C. perfringens* counts and mortality rates as well as to stimulate the innate immune response and to reduce the pro-inflammatory responses in favour of a homeostatic-like response in a broiler *C. perfringens* challenge model [249].

##### Pigs

Unlike poultry, data regarding the potential utilization of postbiotics as alternative to antibiotics in the other monogastric animals are very limited. The administration of postbiotics obtained from *L. plantarum* has been reported to improve the gut health of postweaning piglets in terms of reduced incidence of diarrhoea, increased abundance of lactic acid bacteria, decreased abundance of Enterobacteriaceae, increased production of SCFAs, reduced pH and enhanced villus morphology [250].

##### Fish

A similar scenario can also be highlighted in fish, where the utilization of postbiotics derived from *Lactobacillus* [251,252], *Leuconostoc* [251], *Rhodotorula* and *Cetobacterium* [253] has led to promising results in terms of gut health. In particular, Wu et al. recently observed a positive modulation of the gut microbiota in a hybrid sturgeon fed postbiotic-based diets [253]. The administration of postbiotics has also been recently reported to improve the intestinal microbiota as well as to increase protection against *Lactococcus garvieae* infection in rainbow trout [251,252].

##### Pets

Nowadays, in companion animal medicine, there is a lack of research focused on postbiotics functional metabolism. However, it is commonly recognized that postbiotics are mainly produced by saccharolytic and putrefactive proteolytic fermentations [254]. Saccharolytic fermentation has firstly been reported to produce acetate (60%), propionate (25%)—which served as energy sources and modulated the neuroendocrine system—and butyrate (15%)—that acted as the ideal substrate for colonocyte development, and Na^+^ and Cl^−^ reabsorption [255,256]. Recent studies have also evaluated the functionality of postbiotic molecules produced by putrefactive protein metabolism. In particular, polyamines such as spermidine, spermine, putrescine and cadaverine from arginine and lysine catabolism are capable of slowing down the intestinal epithelial cells senescence and of maintaining the gastrointestinal barrier function through occluding production and cadherin expression [216]. The BCFAs and isobutyrate production starting from valine integration has also been demonstrated to be a compelling energy substrate for the colonocytes in an even more efficient and rapid pathway compared to the butyrate [216]. As Wernimont et al. recently suggested, postbiotics can exert positive effects on numerous diseases such as inflammatory enteropathies, kidney diseases and oral diseases [254]. In conclusion, some of the most recent studies involving probiotic compounds and their impact on intestinal health are reported (Table 3).

## 6. Conclusions

The increasing use and misuse of antibiotics in both human and veterinary medicine has progressively led to the spread of antibiotic resistance issues as well as the frequent onset of intestinal dysbiosis. Nutritional alternatives to the use of antibiotics herein critically summarized and discussed (such as probiotics, prebiotics and postbiotics) show a huge potential in the reduction of the use of antibiotics, since they can prevent or be useful as adjuvants in the treatment of gastrointestinal diseases (where the antibiotics are still considered the primary therapeutic choice) or in the resolution of antibiotic-induced dysbiosis.

## Figures and Tables

**Table 1:** Impact of probiotics on gastrointestinal health: main experimental studies.

Animal Species	Challenging	Probiotic	Dose	Impact on Gut Health	References
Humans	*Campylobacter*	*Enterococcus faecium* LAB SF68	10^8^ CFU three times daily	Treatment of acute diarrhoea in adults	Allen et al. (2010) [156]
Humans	Ulcerative colitis	*Lactobacillus rhamnosus* NCIMB 30174, *Lactobacillus plantarum* NCIMB 30173,* Lactobacil-lus acidophilus* NCIMB 30175 and *Enterococcus faecium* NCIMB 30176	Suspension of barley containing about 10 billion live bacteria	↓Intestinal inflammation ↓Faecal calprotectin levels in the UC patients receiving the probiotic.	Bjarnason et al. (2019) [102]
Humans	*Enterococcus,* *Pseudomonas,* *Enteritis in patients with sepsi*	*B. breve*, *L. casei*	1 × 10^8^ CFU/g, 1 × 10^8^ CFU/g	↑Beneficial bacteria and SCFAs ↓Incidence of enteritis and VAP in patients with sepsis	Shimizu et al. (2018) [157]
Humans	*Staphylococcus aureus* and *Streptocococcus epidermidis*	*Lactobacillus fermentum* CECT5716	1 × 10^10^ CFU/g	↑Breastfeeding duration ↓Antibiotic usage	Bond et al. (2017) [158]
Broiler chickens	*Escherichia coli* O78	*Lactobacillus plantarum* K34, *Bacillus subtilis* MORI 91 and *Clostridium butyricum* M7	2 × 10^8^ CFU/g, 2 × 10^8^ CFU/g, 2.06 × 10^8^ CFU/g	↑total lactobacilli and total lactobacilli-enterococci	Tarabees et al. (2019) [112]
Broiler chickens	*Salmonella* Typhimurium	*Lactobacillus casei* ATTC334, *Bifidobacterium breve* JCM1192 and *Bifidobacterium infantis* BL2416	10^8^ CFU/mL	↓*Salmonella* Typhimurium recovery from caecal tonsils ↑IFN-γ and TNF-α	El-Sharkawy et al. (2020) [113]
Broiler chickens	*Eimeria* spp.	*Enterococcus faecium*	2 × 10^12^ CFU/kg	↓histopathological scores ↓oocyst counts in faecal samples	El-Sawah et al. (2020) [114]
Broiler chickens	*Escherichia coli* K88	*Clostridium butyricum*	2 × 10^7^ CFU/kg of feed	↑TNF-α and IL-4 ↑Vh and ↓Cd ↑amylase, protease and lipase	Zhang et al. (2016) [116]
Broiler chickens	*Salmonella enteriditis*	*Clostridium butyricum*	10^6^ CFU/0.2 mL	↓INF-γ, IL-1β, IL-8 and TNF-α	Zhao et al. (2017) [117]
Broiler chickens	*Clostridium perfringens*	*Clostridium butyricum*	1 × 10^9^ CFU/kg	↓IL-17A ↑Claudin-1 and IL-10 ↓*Clostridium perfringens*	Huang et al. (2019) [118]
Broiler chickens	*Clostridium perfringens*	*Bacillus subtilis* DSM 32315	1 × 10^6^ CFU/g of feed	↑microbial alpha-diversity ↑*Ruminococcus*, Ruminococcaceae and *Bacteroides* ↓*Clostridium perfringens*	Bortoluzzi et al. (2019) [120]
Broiler chickens	*Clostridium perfringens*	*Bacillus subtilis* DSM 32315	2 × 10^9^ CFU/g	↑*Bacillus* spp., Lactobacillaceae, *Lactobacillus salivarius* and *Lactobacillus johnsonii* ↓*Clostridium perfringens*	Whelan et al. (2018) [121]
Broiler chickens	*Campylobacter jejuni*	*Saccharomyces cerevisiae boulardii* CNCM I-1079	1 × 10^9^ CFU/kg	↑Vh ↑*Lactobacillus reuteri* and *Fecalibacterium prausnitzii* ↓*Campylobacter* spp.	Massacci et al. (2019) [122]
Piglets	*Escherichia coli* F4 (K88)	*Lactobacillus rhamnosus* ACTT 7469	10^10^ CFU/day or 10^12^ CFU/day	↓diarrhoea frequency ↓faecal coliforms ↑*Lactobacillus* and *Bifidobacterium* ↓TNF-α, IL-8 and pBD2	Li et al. (2012) [123]
Pigs	*Salmonella enterica* serovar 4	*Lactobacillus rhamnosus* GG	1 × 10^9^ CFU/mL (10 mL/day)	↓Prevotellaceae NK3B31 ↑*Fusobacterium*, *Lactobacillus animalis* and *Propionibacterium* ↓CD3^−^CD19^−^cell subsets	Zhang et al. (2019) [124]
Pigs	*Salmonella* Infantis	*Lactobacillus rhamnosus* GG	1.0 × 10^10^ CFU/day	↑T-bet Activation of STAT3 ↓CCL20 ↓*Salmonella* ↑*Bifidobacterium*	Yang et al. (2017) [125]
Pigs	*Escherichia coli* K88	*Lactobacillus acidophilus*	0, 0.5, 1.0 and 2.0 mL/kg of feed	↑*Lactobacillus* ↓*Escherichia coli* K88	Nordeste et al. (2017) [126]
Piglets	*Escherichia coli*	*Bacillus licheniformis* and *Bacillus subtilis*	3.9 × 10^8^ CFU/day (low dose), 7.8 × 10^8^ CFU/day (moderate dose), 3.9 × 10^9^ CFU/day (high dose)	Low/moderate doses: ↓*Bacteroides uniformis*, *Eubacterium eligens*, *Acetanaerobacterium* and *Sporobacter* ↑*Clostridium, Turicibacter, Lactobacillus gasseri* and *Lactobacillus salivarius* ↑GC number High doses: ↑Proteobacteria ↓GC function	Zhang et al. (2017) [127]
Piglets	*Escherichia coli* F4ac	*Bacillus amyloliquefaciens* DSM25840 and *Bacillus subtilis* DSM25841	1.28 × 10^6^ CFU/g feed	↓Enterobacteriaceae ↑immunity genes ↑amino acid metabolism and utilization	Luise et al. (2019) [128]
Rainbow trout	*Streptococcus iniae*	*Enterococcus casseliflavus*	10^7^ CFU/g of feed, 10^8^ CFU/g of feed, 10^9^ CFU/g of feed	↑lactic acid bacteria and total viable aerobic counts ↑total serum protein and albumin, IgM, C3 complement and lysozyme ↑respiratory burst activity of blood leukocytes	Safari et al. (2016) [129]
Nile tilapia	*Aeromonas hydrophila* and *Streptococcus iniae*	*Rummeliibacillus stabekisii*	10^6^ CFU/g, 10^7^ CFU/g	↑leukocytes and lysozyme ↑IL-1β, TNF-α, TGF-β and heat shock protein 70 ↑*Bacillus* and *Lactobacillus* ↓*Streptococcus* and *Staphylococcus*	Tan et al. (2019) [130]
Nile tilapia	*Aeromonas hydrophila*	*Bacillus velezensis* TPS3N, *Bacillus subtilis* TPS4 and *Bacillus amyloliquefaciens* TPS17	1.0 × 10^8^ CFU/ml	↑nitric oxide, IgM and AKP ↑CAT and SOD ↑Vh, Vw, GC count and muscle thickness ↓*Staphylococcus* and *Aeromonas*	Kuebutornye et al. (2019) [131]
Ornamental fish (*Poecilia latipinna*)	*Aeromonas hydrophila* (BCCM5/LMG3770)	*Bacillus subtilis*-enriched *Artemia*	1 × 10^5^ CFU/mL	↑*Bacillus*	Ahmadifard et al. (2019) [132]
Infant rabbits	Shiga toxin-producing *Escherichia coli* (STEC)	*Lactobacillus casei* strain Shirota	2 × 10^3^ CFU/mL	↑IgA ↓STEC ↓Stx ↓histological damage to the gut mucus	Ogawa et al. (2001) [133]
Rabbits	*Escherichia coli* AZ1	*Lactobacillus casei* SABA6	1 × 10^8^ CFU/mL	↓Eruption of the intestinal epithelial cells ↓Diarrhoea incidence ↑IL-6 and IL-10	Fayyaz et al. (2018) [134]
Dogs	*Aereomonas, psedudomonas*, coliforms	*Lactobacillus fermentum* AD1	3 × 10^9^ CFU/mL	↑Faecal lattobacilli ↓Proteobacteria	Strompfova et al. (2012) [148]
Dogs	IBD	*L. casei*, *L. plantarum*. *L. acidophilus*, *L. delbrueckii* subsp. *Bulgaricus, B. longum*, *B. breve*, and *B. infantis* and *Streptococcus sulivarius* subsp *thermophilus*	112 to 225 × 10^9^ CFU × 10 kg	↑Increases in *Faecalibacterium* spp. and *Lactobacillus* spp.	Rossi et al. (2014) [159]

↑ = increase; ↓ = decrease.

**Table 2:** Impact of prebiotics on gastrointestinal health: main experimental studies.

Animal Species	Challenging	Prebiotic	Dose	Impact on Gut Health	References
Humans	Changes in the faecal microbiota	2′-O-fucosyllactose (2′FL) and/or lacto-N-neotetraose (LNnT)	5, 10 or 20 g per day	↑Bifidobacteria abundance, = abundance beneficial taxa such as Faecali bacterium, Roseburia, Akkermansia or *Lactobacillus*	Elison S. et al. (2016) [180]
Humans	None	Inulin	12 g per day	growth of a limited number of colon bacteria, ↓Bilophila growth	Vandeputte et al. (2017) [209]
Humans	Obesity and associated dysbiosis	Inulin/oligofructose	8 g twice a day	Changes in the gut microbiota correlated in ↓ fat mass, ↓ serum LPS levels and ↓ metabolism (hippurate, lactate and PC)	Dewulf et al. (2013) [166]
Humans	Obesity in children	oligofructose-enriched inulin	8 g per day	↑Bifidobacterium and ↓Bacteroides vulgatus ↓body weight, body fat, percent trunk fat	Nicolucci et al. (2017) [172]
Humans	*Clostridium perfringens*	oligofructose- enriched inulin, *Lactobacillus rhamnosus* and *Bifidobacterium lactis* Bb12	*Lactobacillus rhamnosus* and *Bifidobacterium lactis* Bb12 > log10 CFU/g product oligofructose/inulin 8 g per day	↑bifidobacteria and lactobacilli, ↓*Clostridium perfringens*,	Rafter et al. (2007) [210]
Broiler chickens	*Escherichia coli*	MOS (together with *Saccharomyces cerevisiae*)	0.5%	↓TLR4, NF-κB p65 and IL- 1β ↑IL-10 and occludin ↑Vh and Vh/Cd ↑bacterial diversity	Wang et al. (2016) [188]
Turkey poults	*Salmonella* and *Campylobacter*	MOS and *Saccharomyces cerevisiae* wall (together with a direct-fed microbial)	0.05%	↑villi surface area and Vh/Cd ↓*Salmonella* shedding	Rahimi et al. (2019) [189]
Pullet and layer chickens	*Eimeria* spp.	Whole yeast cell product	0, 0.1 or 0.2%	↓faecal and gut coccidial oocyst counts ↑*Lactobacillus* ↓CD8+ T cell in the cecal tonsils ↓IL-10	Markazi et al. (2017) [190]
Young broiler chickens	*Salmonella enteriditis*	MOS and XOS	1% and 2%	↓*Salmonella enteriditis* counts ↑*Coprococcus*, *Ruminococcus, Enterococcus*, *Clostridium*, *Lactobacillus* and *Roseburia* ↓TNF-α and IFN-γ	Pourabedin et al. (2016) [191]
Broiler chickens	*Salmonella* Typhimurium	XOS (together with *Lactobacillus*-based probiotic and fermented soybean meal)	2%	↓*Salmonella* Typhimurium colonization ↑intestinal lactic acid bacteria ↑mucosal morphology	Jazi et al. (2019) [192]
White Leghorn chickens	*Salmonella* spp.	FOS and probiotics	0.5 or 1%	↓*Salmonella enteriditis*	Luoma et al. (2017) [187]
White Leghorn chickens	*Salmonella* spp.	GOS	1%	↓*Salmonella* Typhimurium	Hughes et al. (2017) [193]
White Leghorn chickens	*Salmonella* spp.	GOS	1%	↓*Salmonella* Typhimurium ↑*Alistipes*, *Lactobacillus reuteri* and Christensenellaceae	Azcarate-Peril et al. (2018) [194]
Broiler chickens	*Escherichia coli* O78	IMO (together with a probiotic mix)	0.5%	↑total lactobacilli and total lactobacilli-enterococci	Tarabees et al. (2019) [112]
Piglets	*Ascaris suum*, *Trichuris suis* and *Escherichia coli* O138:F8	Fructan-rich chicory roots	30%	↓*Ascaris suum* worms ↑*Trichuris suis* worms ↓*Enterobacter* and *Campylobacter* ↑Lachnospiraceae and Bifidobacteria	Jensen et al. (2011) [195]
Pigs	*Trichuris suis*	Inulin	10%	↑IL-13, IL-5 and TFF3 ↓IFN-γ, IL-1α, IL-8 and CXCL9 ↓Proteobacteria and Firmicutes ↑Actinobacteria, Bacteroidetes and Bacteroidetes:Firmicutes ratio ↓pH	Myhill et al. (2018) [196]
Pigs	*Trichuris suis*	Inulin	10%	↑Prevotella ↓Proteobacteria	Stolzenbach et al. (2020) [197]
Pigs	Rotavirus OSU	HMO and GOS-FOS mixture	4 g/L and 3.6 g/L–0.4 g/L	↓diarrhoea duration ↑IFN-γ and IL-10 ↑IgM ↑Lachnospiraceae	Li et al. (2014) [198]
Rainbow trout	*Yersinia ruckeri*	Plant-based diet	Replacement of 10% of fishmeal	↑*Streptococcus*, *Leuconostoc* and *Weissella* counts ↓*Yersinia ruckeri* counts ↓IL-1β and MBL-2	Ingerslev et al. (2014) [199]
Sea cucumber	*Vibrio* spp.	AOS and *Bacillus licheniformis* WS-2	10%	↑immune enzyme activities ↑*Bacillus* and *Lactococcus* ↓*Vibrio*	Wang et al. (2017) [200]
Rabbits	*Eimeria* spp.	*Saccharomyces cerevisiae* cell wall, MOS and dried *Saccharomyces cerevisiae* fermentation solubles	2 g/L	↓faecal oocyst and endogenous stage counts	El-Ashram et al. (2019) [201]
Dogs	faecal fermentative end- products	FOS	0.5%	↑Growth of Bifidobacteria	Pinna et al. (2018) [203]
Cat	*Escherichia coli*	FOS and cellulose	0.05%	↑Bifidobacteria ↓Escherichia coli	Barry et al. (2010) [208]
Cat	*Clostridium perfringens*	Diet with the supplement of FOS and pectin	1% and 2%	↓Clostridium perfringens ↑lactobacilli	Barry et al. (2010) [208]

↑ = increase; ↓ = decrease.

**Table 3:** Impact of postbiotics on gastrointestinal health: main experimental studies.

Animal Species	Challenging	Postbiotic	Dose	Impact on Gut Health	References
Humans	Regulation of intestinal function	*Lactobacillus gasseri* CP2305	1 × 10^10^ bacterial cells per container	↑ F. prausnitzii, ↑ SCFAs	Sawada et al. (2016) [255]
Humans	Viral gastroenteritis (Rotavirus)	Lactoferrin Apo-bLF	50 mg/mL	Inhibition of cytopathic effect In human colon adenocarcinoma (HT-29) cells	Wakabayashi et al. (2014) [256]
Humans	Diverted colorectal mucosa	Sodium butyrate	30 mL of sodium butyrate 600 mmol/Lenema	Endoscopic scores were significantly improved ↑Upregulation of genes associated with mucosal repair	Luceri et al. (2016) [257]
Laying hens	None	*Lactobacillus plantarum* TL1, RS5, RG14, RG11 and RI11	0.6%	↑Lactic acid bacteria ↓Enterobacteriace ↑Propionic acid	Loh et al. (2014) [246]
Broiler chickens	None	*Lactobacillus plantarum* RI11 and RG14	0.3%	↓pH ↑Lactic acid bacteria ↓Enterobacteriaceae ↑Acetic acid	Kareem et al. (2016) [247]
Broiler chickens	None	*Lactobacillus plantarum* RG14	0.15, 0.30, 0.45 and 0.6%	↑Bifidobacteria ↓*Escherichia coli* ↓IFN-γ and LITAF ↑IL-6	Kareem et al. (2017) [248]
Broiler chickens	Heat stress	*Lactobacillus plantarum* RI11, RS5 and UL4	0.3%	↑Vh, ↓Cd and ↑Vh/Cd ↑*Lactobacillus* and Bifidobacteria ↓Enterobacteriaceae, *Salmonella* and *Escherichia coli*	Humam et al. (2019) [245]
Broiler chickens	*Clostridium perfringens*	*Pediococcus acidilactici* B-67717, *Lactobacillus reuteri* B-67718, *Enterococcus faecium* B-67720 and *Lactobacillus acidophilus* B-67701	1 ounce/gallon	↓Lesion scores ↓*Clostridium perfringens* counts	Johnson et al. (2019) [249]
Piglets	None	*Lactobacillus plantarum* TL1, RG11, RI11, RG14 and RS5	0.3%	↓diarrhoea incidence ↑lactic acid bacteria ↓Enterobacteriaceae ↑SCFAs ↓pH ↑Vh	Thu et al. (2011) [250]
Hybrid sturgeon	None	*Rhodotorula* and *Cetobacterium*	5%	↑Firmicutes and *Clostridium* ↓Proteobacteria, Actinobacteria, Chlamydiae and *Lactococcus*	Wu et al. (2020) [253]
Rainbow trout	*Lactococcus garvieae*	*Lactobacillus* and *Leuconostoc*	3%	↑bacterial diversity and richness ↑Firmicutes, *Lactobacillus*, *Enhydrobacter*, *Paracoccus* and *Pseudomonas* ↓Proteobacteria and Fusobacteria	Pérez-Sánchez et al. (2020) [251]
Rainbow trout	None	*Lactobacillus*	3%	↑Tenericutes, Bacteroidetes and Spirochaetes ↓Fusobacteria	Mora-Sánchez et al. (2020) [252]
Dogs	Gut health	bSCFA; isobutyrate (2-methylpropionate)	Substrate from Protein; valine	Source of energy for colonocytes; refeed starved colonocytes with greater efficiency and rapidity than butyrate	Koh et al. (2016) [216]
Dogs	Gut health	SCFA; butyrate	Substrate from Carbohydrate; indigestible polysaccharides	↑Energy substrate for colonocytes; epigenetic modulation; colon electrolyte balance, motility, blood flow	Jackson et al. (2019) [258]

↑ = increase; ↓ = decrease.
